# Is showbiz good for your health?

**DOI:** 10.2471/BLT.15.020715

**Published:** 2015-07-01

## Abstract

Catchy music spots and engaging storylines can help to convey important public health messages, but do they improve our health? Gary Humphreys and Fiona Fleck report.

Ever since the dawn of cinema the entertainment industry has seemed oblivious to the harm done to people’s health by some of the behaviour depicted in its products.

Scenes glamourizing smoking and glorifying all kinds of violence, drink-driving, unsafe sex and substance abuse have long been everywhere in film, television shows and – more recently – on the Internet.

It’s not surprising that public health advocates in the home of the world’s largest entertainment business, the United States of America (USA), have a long tradition of engaging with the entertainment industry.

Not only is there a vast quantity of health-harming imagery and ideas to challenge, but also a powerful channel for ideas. The most popular television shows can reach 20 million people in an hour and, when syndicated, may be watched in as many as 100 countries around the world – so the potential for outreach is huge. But are the ideas these shows promote culturally specific?

Some public health messages may be more relevant to viewers in the USA, but others resonate with viewers in low- and middle-income countries, argues Sandra de Castro Buffington, founding director of the Global Media Center for Social Impact at the University of California, Los Angeles.

She cites as evidence a storyline about HIV infection in a 2001 episode of the *Bold and the Beautiful*, one of the country’s longest running soap operas, which resulted in the highest number of callers that year to the US Centers for Disease Control and Prevention (CDC) HIV/AIDS hotline.

According to a study by Anne O’Leary and colleagues published in the journal *AIDS education and prevention* in 2007, the programme was aired in other countries including Botswana, where viewers’ attitudes to HIV infection apparently changed as a result of the broadcast, helping to reduce stigma attached to the disease.

Getting the message across effectively is one thing, but getting people to change their behaviour is quite another.

In the late 1980s, the Harvard Alcohol Project promoted the idea of the “designated driver” – when individuals take it in turns to drive and not drink – by partnering with the entertainment industry to incorporate the message into television shows *Cheers*, *LA Law* and *The Cosby Show*.

By 1990, surveys showed that 9 in 10 adults were familiar with the designated driver concept and approved of it. 

But, as the authors of a study published in *Journal of the Australasian College of Road Safety* in 2009, note: “The available evidence suggests that while designated driver campaigns can successfully increase the awareness and use of designated drivers, it is less clear whether these programmes lead to a reduction in drink driving and/or alcohol-related crashes.”

The Global Media Center for Social Impact acts as a resource for writers looking to talk with experts in different health fields; takes writers out into the field – whether in the tougher districts of Los Angeles or remote parts of India and South Africa; and organizes conferences and hosts meetings for writers and health experts.

“Often screen writers come to us because they need to verify content with an expert,” de Castro Buffington explains, “but of course we choose the expert, and have a certain amount of influence on what is going to be discussed.”

For example de Castro Buffington arranged a meeting between Atul Gawande, a surgeon at Brigham and Women's Hospital in Boston, and scriptwriters from the television series, *ER* (Emergency Room) on national public television network NBC in 2008.

An accomplished storyteller himself, Gawande regaled the group with tales of the operating room, and got onto the topic of the “surgical checklist”, an initiative he helped to develop with the World Health Organization to reduce the risk of medical error during surgery.

The result: the checklist became the centrepiece of the storyline of an *ER* episode that aired to 10.8 million viewers. “The press in the USA and Canada ran articles about the checklist for days,” de Castro Buffington recalls.

“Not only was there an increase in awareness about the checklist, but the episode was used to persuade surgeons to adopt it, including at a major New York hospital, and the French National Health Authority requested a clip to show at a national stakeholder meeting,” she says.

Beyond simple public health announcements on radio or television, the USA has a long history of incorporating public health messages in film and television storylines.

This form of social marketing – practised by philanthropic foundations among others – can be traced back to the early years of the cinema, according to medical historian Paul Weindling from Oxford Brookes University in the United Kingdom.

“Since the early 20th century foundations have been primarily expert-led, while at the same time pursuing populist strategies,” Weindling says. “Health exhibitions – one of the earliest of which was held in Dresden (Germany) in 1911 – were major public attractions using art works, dramas and later cinema to communicate their messages.”

One of the earliest examples of foundations taking this approach was in 1920, when the Rockefeller Foundation commissioned a (silent) film called *Unhooking the Hookworm* to convey important messages about health risks to the population at large, Weindling notes.

“Foundations developed extensive expertise that could be drawn on to promote outreach and from the 1930s to the 1950s, the Rockefeller Foundation sponsored studies of public impact and mass communication,” Weindling says.

Over the last two decades, partnerships between entertainment and public health in the USA have focused on raising awareness about HIV infection. For example, the California-based Kaiser Family Foundation started its first partnership with a media company in 1997 on a public health information campaign on HIV infection with MTV (Music Television).

“What distinguished Kaiser’s approach from previous public service campaigns is that we looked to media as partners rather than simply a means of distribution,” says Tina Hoff, senior vice president and director of health communication and media partnerships at Kaiser.

“That meant engaging companies – including drawing on their expertise in understanding their audience – in the development of targeted campaigns,” Hoff says.

Since then, Kaiser has collaborated with other entertainment companies including Black Entertainment Television (BET) and Univision Spanish-language broadcaster by providing expertise in public health messages “to ensure they are accurate and by co-producing programming and targeted public service messages,” she says. In 2004, Kaiser launched the Global Media AIDS Initiative with UNAIDS and recently launched the Greater Than AIDS campaign. 

Such initiatives have also come from the US government.

In 1994, the US government started to collaborate on public health information with the entertainment industry and CDC awarded the University of Southern California’s Annenberg School a grant to develop the idea, resulting in the Hollywood Health and Society programme that was led by De Castro Buffington for five years.

“Studies published in peer-review journals show that when you present accurate content in an entertaining format, viewers gain new knowledge and may change their attitudes and behaviour,” says de Castro Buffington.

Studies also show that violence has not only increased in US films and television, but that films featuring violence have been down-rated, so that they are more widely available to younger viewers. So can partnerships with the entertainment industry, for example, challenge the violence shown in film and television shows and, in turn, in real life?

Violence, including domestic violence, was one of the themes of a tour organized by de Castro Buffington of South Africa that focused on community-based solutions. 

 “It was remarkable. These women were telling us how they would stand around the house banging on pots and pans until the husband stopped beating his wife,” says writer and film producer Karen Tenkhoff, who was on the tour, describing a meeting with a group of women in Johannesburg. “It was a way of shaming him and of disrupting what was going on.”

Pot-banging scenes have since been incorporated into the 2012–2013 US television series *Touch*, by writer Carol Barbee, who was also on the tour, and into storylines by the creators of the South African television series *Soul City* and other teledramas that set out to educate and entertain the South African public about HIV infection and other health or social issues.

“Clearly representing women’s empowerment in the face of domestic violence is going to change the way people think about this important public health issue,” Tenkhoff says, adding that “audiences like to watch people doing bad things, including doing things that are bad for people’s health. Still, even these scenarios provide opportunities for education and awareness.”

“*Breaking Bad* showed the dark side of addiction, but also portrayed characters in active recovery,” Tenkhoff says, referring to a recent television drama series about a terminally-ill chemistry teacher who manufactures illicit drugs to secure his family’s financial future.

A few years ago the Bill & Melinda Gates Foundation got involved by providing de Castro Buffington with funding to create a global network of centres. 

She has since opened The Third Eye in India, in collaboration with the Asian Center for Entertainment Education, as a free resource for the Indian film and television industries. She also set up a global centre in Nigeria, in collaboration with Nollywood Labs, to work with the country’s film industry to create storylines that could have a positive impact on health.

For de Castro Buffington, the best way to wield influence over the entertainment industry is not to push a particular message but to inspire television and film writers in Hollywood and other creative centres around the world with great stories from the front line of public health. 

“This is not message placement,” she says. “Writers need to be excited on their own terms to do their best work.” 

**Figure:**
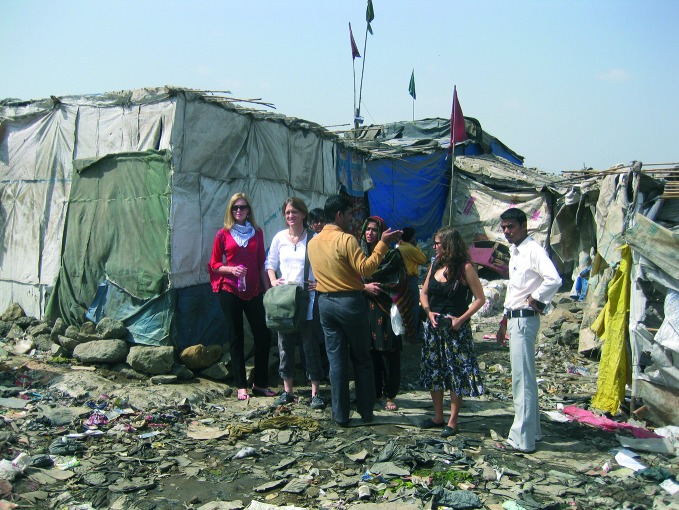
Sandra de Castro Buffington (first on left) and colleagues on a story tour of a Mumbai slum in India.

Courtesy of the Global Media Center for Social Impact.

**Figure:**
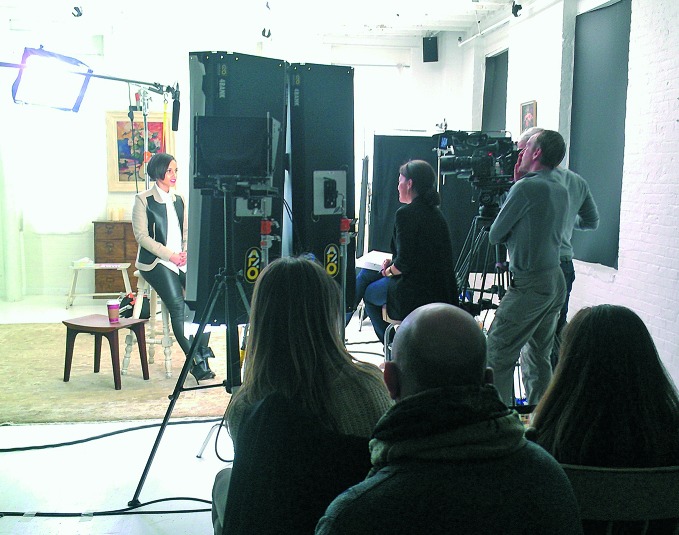
Singer-song writer Alicia Keys and production crew on set recording a public service announcement to launch the Greater Than AIDS campaign in 2013.

Courtesy of the Kaiser Family Foundation

